# Silver nanoparticles incorporated dental restorative resin and its antibiofilm effect

**DOI:** 10.1098/rsos.240915

**Published:** 2024-09-18

**Authors:** Verónica Campos-Ibarra, Andrea Rodríguez-Moreno, Norma Verónica Zavala-Alonso, Luis Octavio Vargas-Sanchez, Marcos Loredo-Tobias, María Elena García-Arreola, Benjamin Aranda-Herrera, Jaime Ruiz-Garcia, Ravichandran Manisekaran

**Affiliations:** ^1^ Interdisciplinary Research Laboratory (LII), Nanostructures and Biomaterials Area, Escuela Nacional de Estudios Superiores Unidad León, Universidad Nacional Autónoma de México, León 37689, Mexico; ^2^ Faculty of Stomatology, UASLP, San Luis Potosi, Mexico; ^3^ Biochemical and Microbiology Laboratory, Faculty of Stomatology, UASLP, San Luis Potosi, Mexico; ^4^ Área de Ciencias de la Tierra, Facultad de Ingeniería, UASLP, San Luis Potosí, Mexico; ^5^ Biological Physics Laboratory, Physics Institute, UASLP, San Luis Potosi, Mexico

**Keywords:** silver nanoparticles, dental resin, antibiofilm, bisacrylic polymer

## Abstract

Dental restoration materials are susceptible to bacterial biofilm formation, which damages the restorations and causes oral health problems. Therefore, to overcome this, silver nanoparticles (AgNPs) are studied widely due to their antimicrobial, anti-inflammatory and healing properties. The purpose of this study was to develop a strategy for incorporating AgNPs onto the surface of bisacrylic resin (Bis) to evaluate its antibiofilm effects using *Streptococcus sanguinis* and *Actinomyces naeslundii*. AgNPs with an average size of 25 nm at two different concentrations were dispersed on the Bis surface (Bis-AgNPs) by mechanical deposition. Ag release was quantified until 7 days of incubation. Bacterial growth was assessed using a viability assay kit and observed using confocal microscopy. The biofilm biomass was quantified using arbitrary fluorescence units. Cell viability was evaluated using an MTT assay. The results showed that Bis-AgNPs significantly inhibited biofilm formation along with a significant difference in the viability of human gingival fibroblasts. The quantification confirmed a decrease in Ag release over time, and elemental mapping showed AgNP penetration up to 10 µm from the surface. Therefore, it was concluded that Bis-AgNPs presented enhanced antibiofilm properties, even at a concentration with no adverse effects. Therefore, this nanocomposite may be a promising alternative for biofilm control in temporary restorative materials.

## 1. Introduction

Maintaining oral cavity health during prosthetic treatment is a major challenge as it is necessary to preserve the microflora equilibrium; thus, it is important to educate the patient regarding good oral hygiene and to select appropriate materials that can restore aesthetics and chewing function. For this purpose, the use of antimicrobial agents, well-designed prosthetic restorations and the selection of highly specialized materials with superior properties have been proposed widely [1–3]. In recent decades, polymeric systems have been among the most commonly used restorative materials in dentistry because of their mechanical resistance, low corrosion in the oral environment, biocompatibility with tissues and optical properties, which make them suitable for patients to recover their functions and aesthetics [4–7]. Bisacrylic resin (Bis) is a material composed of dimethacrylates and filler materials, including bisphenol A-glycidyl methacrylate (Bis-GMA) and urethane dimethacrylate (UDMA). Its formula is commercially independent and is used as a provisional restorative material that has gradually replaced the traditional use of autopolymerizing acrylic resins such as poly (methyl methacrylate) (PMMA) [8–10].

However, provisional restorations are susceptible to bacterial retention and colonization owing to biomaterial surface properties, such as roughness, contact area and electrical charges that facilitate microbial adhesion and growth [11–14]. In addition, microbial communities within the oral cavity vary with more than 700 bacterial species and exist mainly as biofilms on teeth, dentures and mucosal surfaces [15,16]. Biofilm formation at these sites is the main cause of provisional failure, which leads to several oral diseases such as mucosal stomatitis, gingival inflammation and secondary caries, which can lead to medical complications [17,18]. The presence of pathogenic bacteria and the formation of dentobacterial plaques on the surface are related to the adhesion of certain species that act as pioneers and adhere directly to surfaces, including oral *Streptococcus* and *Actinomyces*, among which *Streptococcus* represents >80% of primary colonizers [19,20]. In addition, these colonizers increase the therapeutic procedures and treatment costs [21]. This is aggravated when provisional restorations in the mouth are used for a longer duration, either by treatment duration or postponement of appointments by the patient [22,23].

Therefore, to use provisional restorative materials such as Bis, it is important to avoid or inhibit microbial adhesion and subsequent colonization [24]. Various approaches have been developed to modify the surface properties of these materials by incorporating bactericidal and bacteriostatic agents. Owing to the recent developments in nanotechnology, diverse nanomaterials have been extensively investigated. AgNPs are among the best alternatives, as they have an inherited function of broad-spectrum antimicrobial capacity, which inhibits or prevents the development of resistant bacterial strains through various mechanims [25–27]. The design of devices with the amalgamation of polymeric matrices and nanostructures is a field of extensive research, in which a variety of processing techniques have been implemented to fabricate these nanocomposites [28–31]. Some of the manufacturing procedures include *in situ* polymerization, melt mixing/blending and thin-film coatings are commonly used [31–34]. These procedures are time-consuming, cost-effective and require trained professionals. Thus, it is important to create a simple and rapid technique for coating antimicrobial nanoparticles in order to transfer these technologies to the clinical sector and inhibit microbe-based biofilm infections during prosthetic procedures [35].

Based on our search, we found a few investigations in which AgNPs have been incorporated into common polymers for dental treatment and to evaluate their antimicrobial activity and cytocompatibility [36,37]. For instance, when PMMA is loaded with 1 wt% of graphene-AgNPs, minimal toxicity to human cells and antibacterial activity against *Staphylococcus aureus*, *Streptococcus mutans (S. mutans*) and *Escherichia coli (E. coli*) [38]. In another study, 0.3% of AgNPs incorporated into a common orthodontic adhesive (Bis-GMA/EMA monomers) showed antibacterial activity against *S. mutans* [39]. Finally, AgNPs embedded into an ethylene-vinyl acetate copolymer for mouthguard promoted the bacteriostatic effect of *E. coli*, *Streptococcus sobrinus* and *Porphyromonas gingivalis* [40]. Nonetheless, there are no reports on AgNP coating on Bis resin for provisional restorations with antimicrobial properties.

In this study, a facile strategy was proposed to inhibit the formation of bacterial biofilms by incorporating AgNPs on the polymeric surface bis (Luxatemp^®^) by mechanical deposition. This proof-of-concept methodology has been studied widely using different characterization techniques, antimicrobial activity on the biofilm formation of *Streptococcus sanguinis (S. sanguinis*) and *Actinomyces naeslundii (A. naeslundii*) and the cell viability of the Bis-AgNP-modified resin.

## 2. Material and methods

### 2.1. Synthesis and characterization of AgNPs

The methodology was adopted from [41] with slight modifications. AgNPs were synthesized using 1 mM silver nitrate (Sigma-Aldrich, AgNO_3_) as a precursor, which was dispersed in deionized water with a reducing agent of 10 mg gallic acid (Sigma-Aldrich^®^). The pH of the synthesis solution was adjusted to 11 using 1 M sodium hydroxide (Golden Bell). The entire synthesis was performed under magnetic stirring (IKa^®^ C-MAG HS 7) at room temperature for 30 min. The resulting AgNPs were centrifuged at 4000 rpm for 5 min (Thermo Scientific CL10 centrifuge) and dried in a temperature-controlled oven (Memert^®^) at 80°C for 30 min to obtain NPs in powder form for use in further experiments.

The colloidal dispersion was characterized by transmission electron microscopy (TEM, JEOL JEM-1230 at 100 kV). UV-vis spectra (Multiskan Go, Thermo Scientific) were recorded to confirm the plasmonic band, and the hydrodynamic size was confirmed using dispersed light spectroscopy (DLS, Malvern Nanosizer).

### 2.2. Bis-AgNPs composite preparation and characterization

**Figure 1.**
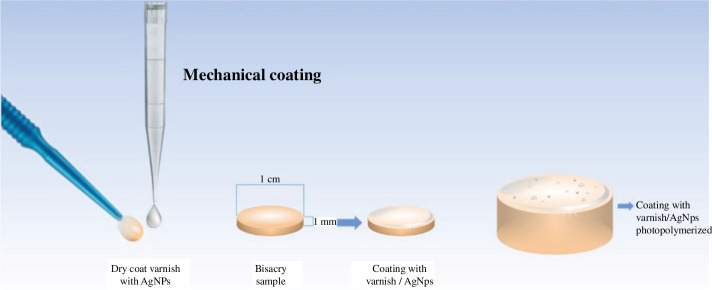
Mechanical deposition of AgNPs to Bis surface using a photopolymerizable varnish. For coating, a microbrush was used with 2 µL of vanish prepared with AgNPs, which was subsequently placed on the disc and polymerized using an LED lamp (Demi™ Plus, Kerr^®^).

Commercial bisacrylic resin (Luxatemp^®^) from DMG was chosen for this study because of its mechanical and colour stability, resistance to bending and abrasion and biocompatibility with oral cavity tissues. Initially, Bis resin was injected into a silicone mould and compressed with a glass tile to obtain a flat surface. Then powdered AgNPs were incorporated into 2 mL of the Dry coat^®^ varnish to obtain a concentration of 0.01 wt% and 0.03 wt%. The deposition was calibrated in 2 μL of the monomer-AgNPs solution and then deposited using a microbrush (Vamasa) to evenly distribute the AgNPs by mechanical coating on the surface of the Bis resin. After the final polymerization phase, the varnish was photopolymerized (figure 1). Controls were prepared using the conventional injection technique, following the manufacturer’s instructions. After polymerization, the discs were washed with ethanol and used.

The morphologies and elemental analyses of Bis-AgNPs were observed using scanning electron microscopy (SEM, Tescan) operated at 1.00 kV equipped with energy-dispersive spectroscopy (EDS). The inductively coupled plasma mass spectrometry (ICP-MS) was used to quantify Ag release after 24, 48, 144 and 168 h in PBS at 37°C under dynamic conditions (MaxQ6000, Thermo Scientific). From different intervals, 1.5 mL was collected in an Eppendorf tube, and the replaced solution was balanced with a similar volume of PBS solution to maintain the same volume throughout the experiment.

### 2.3. Antibiofilm assay

#### 2.3.1. Bacterium inoculum preparation

*S. sanguinis* (ATCC 10556) and *A. naeslundii* (ATCC 27039) strains used in this study were grown on brain/heart infusion agar plates (BBL; Becton, Dickinson, NJ, USA) at 37°C for 48 h under microaerophilic conditions (5% O_2_, 10% CO_2_). After incubation, the microbial growth of each strain was collected and inoculated in 15 mL Falcon tubes containing 5 mL of Tryptone Soya Broth (TSB, Sigma-Aldrich) and incubated again at 37°C for 16 h. A smear was then made to corroborate the formation of the desired microorganisms employing Gram staining and was observed under an optical microscope at 40X magnification. The broth culture was centrifuged, washed and vortexed twice. From the obtained suspension, an initial stock was prepared at an absorbance of 0.5 McFarland at a wavelength of 600 nm in a spectrophotometer. This absorbance corresponded to an approximate concentration of 1–5 × 10^8^ bacterial cells/ml. Finally, 15 µL of *A. naeslundii* and 86 µL of *S. sanguinis* were suspended in 1 mL of enriched broth to obtain the final inoculum.

#### 2.3.2. Biofilm formation

Resin samples were washed and disinfected with ethanol in an ultrasound bath (Branson 2510). They were then dried and placed in Petri dishes for sterilization under UV irradiation for 20 min. This procedure was performed using gloves and tweezers to avoid external contamination. Polymeric discs were placed in 24-well plates and inoculated with 1 mL of each bacterium at a 50/50 ratio. The samples were incubated at 37°C for 7 days in a shaker to promote biofilm formation, and the culture medium was replaced every 24 h.

#### 2.3.3. Confocal microscopy

After incubation time had elapsed, the samples were washed with PBS, stained with the LIVE/DEAD Bacterial Viability Baclight system (Invitrogen), and observed under a confocal laser scanning microscope (CLSM; Leica DMI 4000 B) at an excitation wavelength of 480 nm and two emission wavelengths. During observation, images of the control and experimental discs were obtained by observing one field of the inoculated surface of each disc at 40X magnification for qualitative evaluation. Subsequently, total quantification was performed for each image by measuring arbitrary fluorescence units (AFU).

### 2.4. Cytotoxicity test

#### 2.4.1. Cell culture growth

Human gingival fibroblast (HGF) was obtained from gingiva tissues, during the surgery of third molar extraction from a 25 year-old patient with a previously signed consent form and approved protocol by the Bioethics Committee of the ENES-Leon, UNAM. The cells were seeded onto a 10 cm culture dish and cultured in modified Eagle’s medium (MEM, Life Technologies, Gibco, USA) supplemented with 10% heat-inactivated foetal bovine serum (FBS, Life Technologies, Gibco), 100 UI/ml penicillin (Sigma-Aldrich) and 100  mg/ml streptomycin (Life Technologies, Gibco). Primary cultures were incubated at 37 °C in a humidified atmosphere containing 5% CO_2_ until the cell population reached 80% confluency. The cells were harvested by treatment with 0.05% trypsin-EDTA-2Na in PBS and subcultured. The cells were then subcultured in Dulbecco’s modified Eagle’s medium (DMEM, Life Technologies, Gibco) supplemented with 10% FBS, 1% Glutamax (Life Technologies, Gibco) and 2% penicillin and streptomycin.

#### 2.4.2. MTT assay

To evaluate the viability of Bis-AgNPs, cells (6 × 10^4^ cells/ml) were inoculated into 24-microwell plates and incubated for 48 h to achieve complete cell adherence. The samples were resuspended in DMEM for indirect contact and further incubated for 24 h. The cells were inoculated with the samples in the same manner as for direct contact. The relative number of viable cells was determined using the MTT method. Briefly, the culture medium was replaced with MTT (0.2 mg/ml) dissolved in DMEM, and cells were incubated for 4 h at 37°C. After replacing the medium, the formazan product was dissolved in DMSO and the absorbance at 570 nm was measured using a UV-vis spectrophotometer (Multiskan^®^ GO, Thermo Scientific). The effect of each nanocomposite was calculated in triplicate from three independent experiments.

#### 2.4.3. Statistical analysis

Statistical analysis was performed using one-way analysis of variance (ANOVA), and comparisons between groups were confirmed with post hoc Tukey Honestly Significant Difference (HSD) with the software RStudio (2023.06.1 Build 524).

## 3. Results

### 3.1. AgNPs characterization

**Figure 2.**
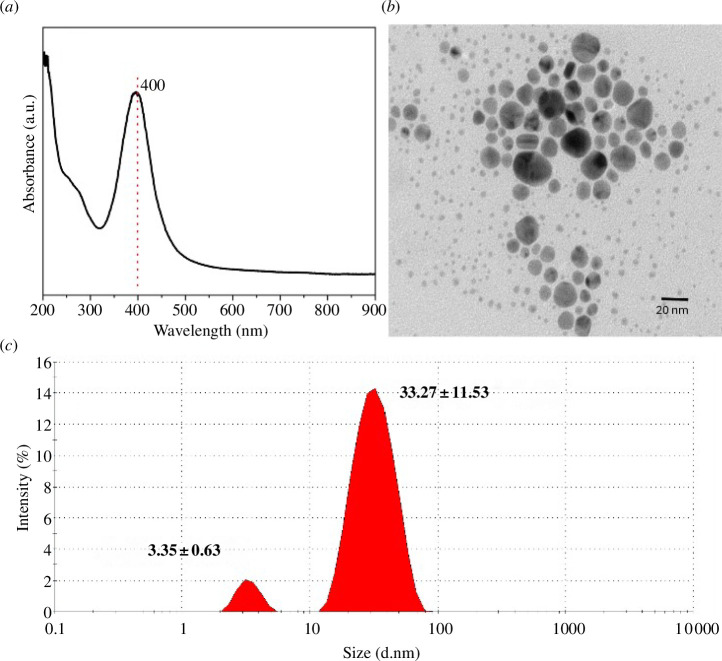
(*a*) UV-vis absorption spectrum of AgNPs, (*b*) TEM image showing a particle size of 2–20 nm and (*c*) DLS analysis with an average hydrodynamic size of 3.35 nm and 33.27 nm.

The AgNPs were initially characterized to confirm the formation of nanoparticles, which can be suitable for further applications. The colloidal solution was analysed using UV-visible spectroscopy to visualize the characteristic surface plasmon peak of AgNPs [41] which exhibited a sharp band at a maximum of 400 nm (figure 2*a*). This band corroborates the formation of AgNPs and other reported works [42,43]. The morphology was analysed using TEM, as shown in figure 2*b*. The synthesized nanoparticles had a size distribution of 2–20 nm and exhibited a pseudospherical shape. Finally, the hydrodynamic size was measured using DLS and observed a bimodal distribution of 3.35 ± 0.63 nm and 33.27 ± 11.53 nm which corroborates with the TEM analysis (figure 2*c*).

### 3.2. Nanocomposite characterization

**Figure 3.**
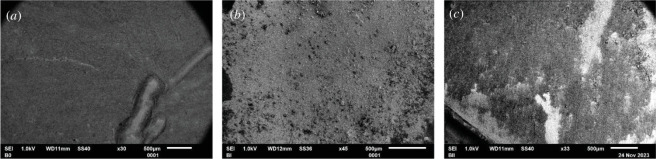
The topography of the disc surface was compared by SEM. In (*a*), bis controls, (*b*) and (*c*) show the topography of 0.01 and 0.03 wt%, respectively.

From the elemental analysis by EDS, the presence of Ag on the surface was confirmed in all groups, except the control samples. Figure 3*a–c* shows obvious differences between the groups. For instance, the control sample surface was smooth, whereas the AgNP-treated resin showed rough surfaces and porosity. Bis resin samples have an average Ag% of 0.4 ± 2.1 in the case of 0.01 wt%, and Bis-AgNPs 0.03 wt% sample showed an Ag% of 1.3 ± 2.9. The experimental groups, the results of which are shown in table 1, were also evaluated transversely 10 µm from the surface and checked at three different points to obtain the average Ag% in the disc.

**Table 1.** Elemental analysis of AgNPs-coated Bis resin.

samples	control	Bis-AgNPs 0.01 wt%	Bis-AgNPs 0.03 wt%
**elemental %**	0	0.4 ± 2.1	1.3 ± 2.9

### 3.3. Ag release

Ag release from the nanocomposite was studied at different time intervals (24, 48, 144 and 168 h), as shown in table 2. The average weight of Bis resin disc without AgNPs was approximately 107.8 mg, which is incorporated with 15.6 ppm for the 0.01 wt% and 31.2 ppm for the 0.03 wt%. Bis-AgNPs (0.01 wt% and 0.03 wt%) have the highest release at 24 h with 44.94 and 49.54 ppb, respectively. The release of Ag from the samples was continuous and decreased after 7 days, but from the second day, the release speed was between 0% and 0.03%, possibly due to a less/loss of contact with the aqueous medium, which affects Ag release.

**Table 2.** Analysis of Ag release in nanocomposite Bis-AgNPs.

time (h)	Bis-AgNPs 0.01%			Bis-AgNPs 0.03%			control
	Ag release (ppb)	total Ag (%)	release speed (µg/día)	Ag release (ppb)	total Ag (%)	release speed (µg/día)	Ag release (ppb)
**24**	44.94 ± 3.63	0.24	44.94	49.54 ± 9.65	0.13	49.54	0.1 ± 0.000
**48**	40.29 ± 3.18	0.03	20.14	45.94 ± 3.76	0.01	22.97	<0.01
**144**	36.54 ± 3.96	0.02	5.09	44.47 ± 5.44	0	7.4	<0.01
**168**	34.64 ± 9.45	0.01	4.9	37.53 ± 6.27	0.02	5.36	0.012 ± 0.003

### 3.4. Antibiofilm effect

**Figure 4.**
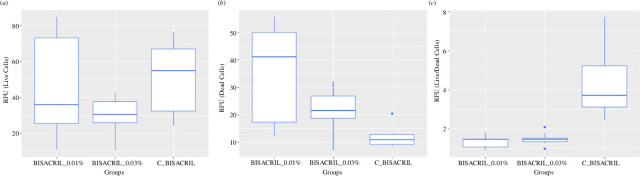
Graphs show the inhibitory effect of nanocomposite obtained by the mechanical deposition method. (*a*), (*b*) and (*c*) show the biofilm formation behaviour of *S. sanguinis* and *A. naeslundii* on the Bis-AgNPs using two concentrations (0.01 wt% and 0.03 wt%) and compared with the control.

The inhibitory effect of the AgNP coating on the resin samples was tested on the biofilms of *S. sanguinis and A. naeslundii*, as shown in figure 4. There was a clear difference between the growth of biofilms in the control group and the treated samples. The graphs are presented in the following order: live cells (figure 4*a*), dead cells (figure 4*b*) and live/dead ratio (figure 4*c*).

**Figure 5.**
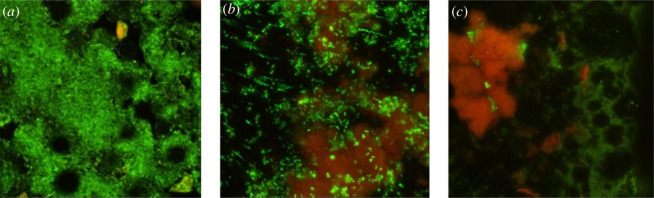
Images (*a*), (*b*) and (*c*) were obtained from the resin surface. (*a*) The formation of biomass was observed on pure resin with high fluorescence. Samples with 0.01 wt% (*b*) coatings show agglomerates of bacteria and red foci, (*c*) 0.03 wt% samples had red foci and little fluorescence, which suggests a greater antimicrobial effect.

**Figure 6.**
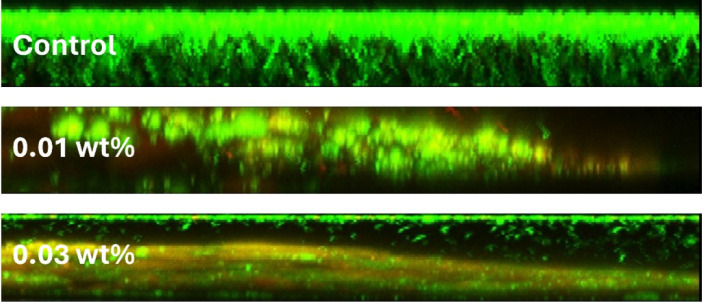
Cross-sections of the biofilm observed by CLSM showed homogeneous growth in the control resin, while in the experimental groups, disorganization of the biomass and red dots were observed, indicating damaged microorganisms and inhibition of the surface.

Bis-AgNPs samples with 0.01 wt% and 0.03 wt% coatings show agglomerates of bacteria which are observed from red foci. However, the entire surface of the 0.03 wt% samples had red foci, suggesting it to be a greater antimicrobial material than the 0.01 wt% samples that were identified as coccoid agglomerates with little fluorescence (figure 5*a–c*). Analysis of the arbitrary fluorescence units (AFU) obtained from the CLSM showed discrete differences among the groups. The control and experimental groups were compared using the non-parametric Kruskal–Wallis test with a post hoc test. There were no statistical differences between the groups (*p* = 0.3) for live cells between the control groups, and the group treated with 0.01 wt% AgNPs had a greater number of dead cells than the control group and even the group treated with 0.03% AgNPs (*p* = 0.02). The transverse section of the biofilm formed on the resin surface is shown in figure 6.

### 3.5. Cytotoxicity of Bis-AgNPs

**Figure 7.**
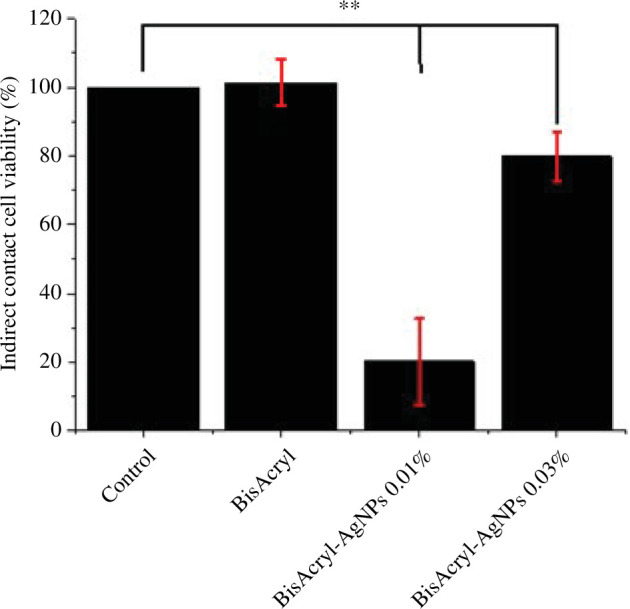
Histogram of indirect contact of composite samples with HGF. The results are significant at *p *< .00001 (**) compared with the pristine resin.

**Figure 8.**
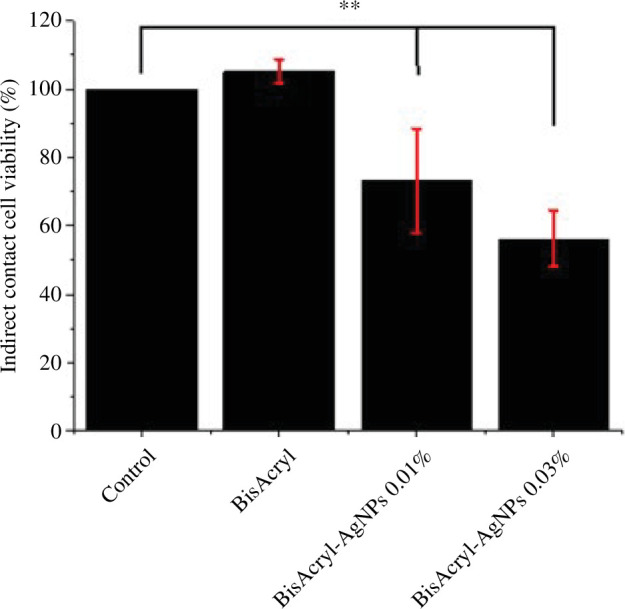
Histogram of direct contact of the composite samples with HGF. The result was significant at *p *< .00001 (**) compared with the resin without AgNPs.

The cytotoxicity of Bis-AgNPs at two different concentrations (0.01 wt% and 0.03 wt%) was assessed by subjecting HGF cells to the resins for indirect (figure 7) and direct (figure 8) contact. The mechanical deposition of AgNPs onto the surface of the Bis resin at both concentrations (0.01 wt% and 0.03 wt%) was significantly different from that of the pristine resin. Various concentrations of AgNPs produced a minor decline in cell viability (more than 50%) when compared with that of the pristine polymer in the case of direct contact. However, in the indirect contact, it is quite surprising to observe that 0.01 wt% of AgNPs with resin showed major toxicity with viability of only 20% cells. However, it is not possible to conclude why this effect requires more extensive study. Therefore, in general, we observed negligible toxicity to cells; however, it mainly depended on the type of contact with the cells.

## 4. Discussion

The development of polymeric biomaterials with Ag or other metal nanoparticles involves several techniques, such as emulsion-based micelle formation, incorporation by aerosols, *in situ* polymerization, laser ablation and mixing solutions [31,32,44,45]. In this study, AgNPs were incorporated using an intermediate varnish by mechanical deposition onto bisacrylic resin, which is commonly used in oral rehabilitation to inhibit the formation of bacterial biofilms. In addition, AgNPs were incorporated at low doses, preventing biofilm formation by *S. sanguinis* and *A. naeslundii*, the first colonizers in the oral cavity [46].

The characterization of the colloidal solution showed a hydrodynamic average size of 40 nm, while the TEM micrograph showed particles of 2−20 nm. Wu *et al.* reported that 2 nm AgNPs showed the best antimicrobial activity compared with sizes of 12 and 32 nm, against *E. coli* and *S. aureus* [47]. However, the antimicrobial activity of AgNPs can produce a wide effective range, ranging from 2 to 50 nm, when friendly syntheses are used [48,49], as the synthesis harnesses in this study. In addition to size, shape plays an important role in the antimicrobial efficacy of AgNPs [50]. Particles in close contact with the bacterial surface show higher activity where some spherical shapes are included, such as those observed in this study by TEM, although those with a cubic, prismatic or hexagonal facet are described as having greater surface energy and higher antibacterial activity for the sharpest ones [51,52]. Meanwhile, the nanocomposite was examined by SEM in each of its concentrations and compared with the control evident differences due to the incorporation of a varnish with silver nanoparticles. Perchynok *et al*. modified a bisacrylic resin with chitosan and nanodiamonds with homogeneous mixes, where no significant differences in the SEM image were observed when compared with the control group [53].

On the other hand, microbial biofilms are a natural form of bacterial organization with a structure embedded in an extracellular polymeric matrix that gives them the ability to tolerate antibiotics and less susceptibility to disinfection methods [54,55]. In the oral cavity, the impact of a biofilm is on the local and systemic health of patients, producing persistent chronic infections, and is a focus of infection for areas away from the biofilm. Therefore, it is important to prevent biofilm formation on dental polymer surfaces [56]. The results of this *in vitro* investigation showed that the biomass of live cells was greater on the 0.01% Bis-AgNPs than on the 0.03 wt% coating with the Dry coat intermediate material, presenting a greater inhibitory effect on the biofilm. The AgNPs synthesized and incorporated into Bis resin by this technique provided an enhanced effect that prevented the growth of *S. sanguinis* and *A. naeslundii* biofilms, as long as a sufficient amount of AgNPs was deposited on the material surface, demonstrating a dose–response relationship. Furthermore, in the CLSM images of the control group, it was observed that when the biofilm reached a certain growth stage, conglomerates and coccoid groupings were formed, including *S. sanguinis* and bacillus-shaped groupings of *A. naeslundii*, which indicated that an increase in the metabolic activity of the bacteria was created, and it became more diverse as it developed. It should be noted that in the Bis resin experimental groups, more dispersed cells were observed in different degrees of viability, with reduced metabolism.

There are no previous reports on the incorporation of AgNPs on Bis resin surfaces, but there are few studies on the effect of glaze and chlorhexidine in resins to provide antiseptic activity to provisional restorations [57,58]. Therefore, our results confirmed that at low doses of AgNPs, an antimicrobial effect was observed. Furthermore, previous reports indicated that a low Ag release was evaluated for 8 and 30 days (420 and 314 ppb) and a lower Ag release was evaluated for 7 days in PMMA; the results obtained here are 84% below those reported for the 0.01% and 96% for the 0.03% groups. Bis resin has a heterogeneous chemical composition formed by an organic and inorganic matrix and bonding agent [59]. The polymer chains may hold and stabilize the AgNPs, allowing for the controlled release of Ag^+^ ions, which inhibits the growth of microorganisms [60]. This low release rate may maintain antimicrobial efficacy over time as a source of ions or particles, causing excessive toxicity to human cells or other organisms. However, the amount of Ag released was well below the values reported *in vitro* and *in vivo* toxicity and inflammation tests, which are in the range of 4–200 ppm [61–63]. In addition, the low release of Ag may suggest that its effect is due to direct contact with the surface, possibly affecting the Gibbs free energy or matrix of exopolysaccharides of bacteria during the early stages of colonization, thereby inhibiting growth [46,64] (65). These promising results suggest that AgNPs can be used to inhibit biofilm formation on provisional restorative materials. However, certain limitations apply to immunocompromised patients, where prevention of infections is necessary.

## 5. Conclusions

In conclusion, the microbrush-based mechanical coating with the incorporation of AgNPs is an effective, simple and rapid strategy to inhibit biofilm formation on the surface of Bis resin, with the advantage of being effective even at very low concentrations (0.01%) of the total weight for dental applications. In addition, the polymeric surface has a lower Ag release over time and shows good cytocompatibility with human gingival fibroblasts when it comes to direct contact. Therefore, we are currently conducting *in vivo* studies to verify these beneficial and in-depth effects. Thus, it is concluded that the fabrication of these types of nanocomposites can have huge potential in the treatment of restorative materials, thereby improving the patient’s life.

## Data Availability

Experimental data related to this article can be accessed from the Dryad Digital Repository [66].
